# Administering Multiple Injectable Vaccines During a Single Visit—Summary of Findings From the Accelerated Introduction of Inactivated Polio Vaccine Globally

**DOI:** 10.1093/infdis/jix054

**Published:** 2017-07-01

**Authors:** Samantha B. Dolan, Manish Patel, Lee M. Hampton, Eleanor Burnett, Daniel C. Ehlman, Julie Garon, Emily Cloessner, Elizabeth Chmielewski, Terri B. Hyde, Carsten Mantel, Aaron S. Wallace

**Affiliations:** 1 Global Immunization Division, Centers for Disease Control and Prevention, and; 2 Emory University School of Medicine, Atlanta, and; 3 Task Force for Global Health, Decatur, Georgia;; 4 Johns Hopkins University, Baltimore, Maryland; and; 5 Department of Immunization, Vaccines, and Biologicals, World Health Organization,Geneva, Switzerland

**Keywords:** Polio eradication, systematic review, vaccine administration, vaccine introduction.

## Abstract

**Background.:**

In 2013, the World Health Organization’s (WHO’s) Strategic Advisory Group of Experts (SAGE) recommended that all 126 countries using only oral polio vaccine (OPV) introduce at least 1 dose of inactivated polio vaccine (IPV) into their routine immunization schedules by the end of 2015. In many countries, the addition of IPV would necessitate delivery of multiple injectable vaccines (hereafter, “multiple injections”) during a single visit, with infants receiving IPV alongside pentavalent vaccine (which covers diphtheria, tetanus, and whole-cell pertussis; hepatitis B; and *Haemophilus influenzae* type b) and pneumococcal vaccine. Unanticipated concerns emerged from countries over acceptability of multiple injections, sites of administration, and safety. We contextualized the issues surrounding multiple injections by documenting concerns associated with administration of ≥3 injections, existing evidence in the published literature, and findings of a systematic review on administration practices and techniques.

**Methods.:**

Concerns associated with multiple-injection visits were documented from meetings and personal communications with immunization program managers. Published literature on the acceptability of multiple injections by providers and caregivers was summarized, and a systematic review of the literature on administration practices was completed on the following topics: spacing between injection sites (ie, vaccine spacing), site of injection, route of injection, and procedural preparedness. WHO and United Nations Children’s Fund data from 2013–2015 were used to assess multiple-injection visits included in national immunization schedules.

**Results.:**

Healthcare provider and caregiver attitudes and practices indicated concerns about infant pain, potential adverse effects, and uncertainty about vaccine effectiveness with multiple-injection visits. Published literature reinforced the record of safety and acceptance of the recommended schedule of IPV by the SAGE, but the evidence was largely from developed countries. Parental acceptance of multiple injections was associated with a positive provider recommendation to the caregiver. Findings of the systematic review identified that the intramuscular route is preferred over the subcutaneous route for vaccine administration and that the vastus lateralis muscle is preferred over the deltoid muscle for intramuscular injections. Recommendations on vaccine spacing and procedural preparedness were based on practical necessities, but comparative evidence was not identified. During 2013–2015, 85 countries added IPV to their immunization schedules, 46 (55%) of which adopted a schedule resulting in 3 injectable vaccines being administered in a single visit.

**Conclusion.:**

The multiple-injection experience identified gaps in guidance for future vaccine introductions. Global partner organizations quickly mobilized to assess, document, and communicate the existing global experience on multiple-injection visits. This evidence-based approach provided reassurance to opinion leaders, health workers, and professional societies, thus encouraging uptake of IPV as a second or third injection in an accelerated manner globally.

In 2013, the Global Polio Eradication Initiative (GPEI) launched the Polio Eradication and Endgame Strategic Plan 2013–2018 to comprehensively address both polio eradication and endgame objectives [1]. Since 2006, a substantial proportion of polio cases have been caused by circulating vaccine-derived polioviruses, prompting the GPEI to advance its endgame strategy of withdrawing oral polio vaccine (OPV) [2]. While eradication activities for poliovirus types 1 and 3 continue, naturally occurring type 2 wild poliovirus has not been detected since 1999 and was declared eradicated in 2015. Thus, the first phase of the endgame called for removal of the type 2 component of OPV through a globally synchronized switch from trivalent OPV (tOPV) to bivalent OPV (bOPV), which contains poliovirus types 1 and 3, in routine immunization programs. In preparation for the switch in April 2016, the GPEI called for all OPV-using countries to introduce inactivated polio vaccine (IPV), which contains all 3 poliovirus serotypes, to maintain high levels of population immunity to poliovirus type 2. Therefore, 126 countries that were only using OPV to immunize children against polio had to introduce at least 1 dose of IPV as recommended after the GPEI decision by the World Health Organization’s (WHO’s) Strategic Advisory Group of Experts on Immunization (SAGE) [3]. To balance the need to protect children from type 2 poliovirus at an early age with the fact that the immunogenicity of IPV increases with age, a single dose of IPV would be administered to children 14 weeks of age, alongside other routine injectable vaccines, particularly pentavalent vaccine (diphtheria, tetanus, and whole-cell pertussis [DTP]; hepatitis B [HepB]; and *Haemophilus influenzae* type b [Hib]) and pneumococcal conjugate vaccine (PCV) [4].

The accelerated introduction of IPV globally between 2013 and 2016 posed many challenges for GPEI partners. In 2014, the managers of multiple countries’ national immunization programs expressed concerns during meetings and country consultations about the administration of an additional vaccine injection, including concerns about healthcare provider training as well as acceptance of the new vaccine schedule by both providers and child caregivers. While almost all countries had introduced new vaccines over the previous 15 years, these introductions were often facilitated by the use of new combination vaccines such as pentavalent vaccine [5]. In 2014, most countries did not have ≥3 injectable vaccines scheduled during a single visit (Table 1). Combination vaccines that contained IPV were available at substantially higher prices than standalone IPV; none were WHO prequalified nor eligible for procurement by GPEI, UNICEF, and its partners at Gavi, the Vaccine Alliance [6]. The perceived problems posed by the additional injection from IPV were compounded by the fact that many countries had recently introduced or planned to introduce PCV. Concerns about an additional vaccine injection associated with IPV introduction were anticipated by GPEI, but were not considered critical enough to warrant policy changes before country discussions and regional workshops on IPV introduction. SAGE policy and working group discussions on the endgame did not mention possible implications of the additional IPV injection [3, 7–11]. As such, the evidence base, communications, and operational recommendations surrounding delivery of multiple injectable vaccines (hereafter, “multiple injections”) during a single visit were insufficiently developed to address countries’ concerns.

**Table 1.** Multiple Injectable Vaccines During a Single Visit and Inactivated Polio Vaccine Use Among National Vaccination Schedules for Children Aged 0–2 Years, by World Health Organization (WHO) Region, 2014 and 2015

Variable	WHO Region and Year													
	Overall (n = 194)		AFR (n = 47)		AMR (n = 35)		EMR (n = 21)		EUR (n = 53)		SEAR (n = 11)		WPR (n = 27)	
	2014	2015	2014	2015	2014	2015	2014	2015	2014	2015	2014	2015	2014	2015
*No. of injectable vaccines recommended during 1 visit*														
**2 injectable vaccines**	167 (86)	178 (92)	42 (89)	46 (98)	27 (77)	29 (83)	21 (95)	21 (100)	44 (83)	45 (85)	8 (73)	11 (100)	26 (96)	26 (96)
**3 injectable vaccines**	50 (26)	85 (44)	1 (2)	21 (45)	13 (37)	16 (46)	7 (33)	13 (62)	18 (34)	18 (34)	1 (9)	1 (9)	10 (37)	16 (59)
**4 injectable vaccines**	16 (8)	17 (8)	0 (0)	0 (0)	6 (17)	6 (17)	3 (14)	3 (14)	4 (8)	4 (8)	0 (0)	0 (0)	3 (11)	3 (11)
**5 injectable vaccines**	5 (3)	5 (3)	0 (0)	0 (0)	0 (0)	0 (0)	0 (0)	0 (0)	5 (9)	5 (9)	0 (0)	0 (0)	0 (0)	0 (0)
*Percentage of vaccination visits during which specified no. of injectable vaccines were delivered* ^*a*^														
**1 injectable vaccine**	51	46	45	41	57	53	40	37	53	51	71	54	53	42
**2 injectable vaccines**	39	41	54	51	28	30	50	48	32	35	27	42	36	43
**3 injectable vaccines**	7	10	1	9	11	14	7	11	9	9	2	3	9	12
**4 injectable vaccines**	2	2	0	0	4	4	3	4	2	2	0	1	2	2
**5 injectable vaccines**	0	1	0	0	0	0	0	0	2	2	0	0	0	0
*IPV in vaccination schedule*	76 (39)	151 (78)	1 (2)	25 (53)	7 (20)	28 (80)	11 (52)	18 (86)	43 (81)	46 (87)	1 (9)	9 (82)	13 (48)	25 (93)

Data are no. (%) of countries, unless otherwise indicated. Data are from the WHO/United Nations Children’s Fund [12] Joint Reporting Form process for national immunization schedules during 2014 and 2015 and the Global Polio Eradication Initiative [13] reporting mechanisms for IPV introduction.

Abbreviations: AFR, African Region; AMR, Region of the Americas; EMR, Eastern Mediterranean Region; EUR, European Region; IPV, inactivated polio vaccine; SEAR, South-East Asian Region; WPR, Western Pacific Region.

^a^Data are average percentages across countries in a given WHO region.

In the absence of existing guidance from the SAGE, the WHO provided the following provisional recommendations regarding the operational aspects of administering multiple injections at a single visit for IPV, PCV, and pentavalent vaccines. First, IPV (nonadjuvanted) can be given intramuscularly or subcutaneously, but because of reduced reactogenicity and easier administration, the WHO recommends the intramuscular route. Second, for intramuscular injections in infants <15 months of age, the deltoid injection site (ie, the upper arm) should not be used, because of its inadequate muscle mass. Third, when 3 intramuscular injections are scheduled simultaneously in children <15 months of age, it is safe and acceptable to give 2 injections in the same thigh. The WHO recommendation is injection of PCV plus IPV, separated by 2.5 cm, in one thigh and injection of DTP-HepB-Hib in the other thigh.

During regional meetings and personal communications, national immunization program managers expressed concerns about the increase in the number of injectable vaccines given to infants with IPV introduction (authors’ unpublished data; Figure 1). Some of the expressed concerns about healthcare providers’ possible unwillingness to follow a new schedule, caregivers’ possible refusal to allow children to receive all vaccines because of fears of increased pain, and perceived safety problems from an increased number of injections. Such refusals could result in lower immunization coverage. National immunization program managers from some low-income countries also voiced concerns that, because of a high prevalence of undernourishment and prematurity, many infants in their countries might have inadequate muscle mass for 2 vaccines to be administered in the same limb. National immunization technical advisory groups and immunization program managers also debated which vaccines to administer in which limb; whether IPV should be administered subcutaneously or intramuscularly; the physical spacing between 2 vaccine injections; the order of vaccine administration; the need for longer immunization visits, to allow enough time to administer all vaccines; techniques to reduce pain associated with multiple injections; how to assess whether a particular vaccine had caused a given reported adverse event following immunization; and the possibility of adding additional visits to their recommended immunization schedules. In some cases, these discussions also affected countries’ plans to introduce PCV. These early communications demonstrated to IMG partners that countries were struggling with the complexities of multiple injections, particularly given the short timeline of introduction for IPV, a situation that called for global expert guidance, resources, and communications.

**Figure 1.**
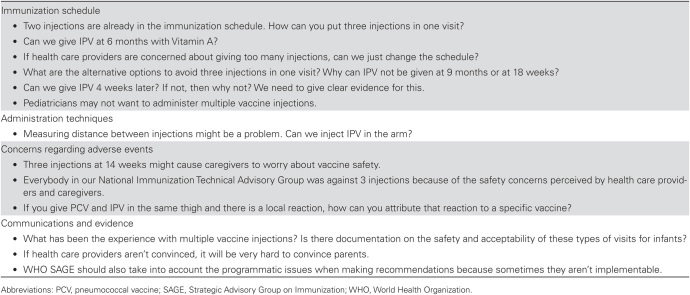
Examples of Concerns Expressed About Increasing the Number of Injectable Vaccines Given to Infants, From Regional and Country Inactivated Polio Vaccine (IPV) Workshops and Advisory Group Meetings

To address concerns related to IPV introduction, GPEI’s Immunization Systems Management Group (IMG) developed a multipronged strategy that included an expert review of the current evidence base, identifying and filling the gaps in evidence, and proactively communicating the findings to address country-specific concerns. In April 2015, SAGE reviewed the published and gray literature on healthcare provider and caregiver attitudes and practices regarding multiple injections; the safety and immunogenicity of administering IPV, PCV, and pentavalent vaccines during a single visit; and techniques for administering multiple injections. We review the multiple injections experience as it evolved during the implementation of the endgame strategy. We report results on the following topics: (1) provider and caregiver acceptance of multiple injections, (2) available evidence for guidelines when administering multiple injections, (3) communication messages on vaccine safety and acceptability, and (4) prevalence of recommended visits involving >1 vaccine injection during the 2013–2015 global IPV introduction.

## METHODS

The methods used for each topic varied. The systematic review methods used for provider and caregiver acceptance are described in detail elsewhere [15].

For guidelines on administering multiple injections, we reviewed evidence from both the peer-reviewed and gray literature (ie, literature not published by academic or commercial entities) that pertains to the recommendations on administering multiple injections in a single visit, with a focus on the administration of the most common Expanded Program on Immunization (EPI) vaccines for infants. Articles were identified through Medline (PubMed) and Embase databases. Five individuals reviewed a unique set of the resulting articles and abstracted relevant information, using a standardized Microsoft Excel–based data collection tool. In addition to this preliminary review, individual published and gray literature reviews were conducted for topics about which little or no peer-reviewed literature was found. Experts were contacted for comments on the topics and additional articles to review if no evidence was identified. Multiple-injection guidance documents and immunization guides were reviewed from selected national immunization programs, and other organizations were included if they were available online in English, Spanish, or French. The following sources were used to identify gray literature: Grey Literature Report (available at: http://www.greylit.org), OpenGrey (available at: http://www.opengrey.eu), the Norwegian Satellite of the Cochrane Effective Practice and Organisation of Care Review Group (available at: http://www.epocoslo.cochrane.org), Google (available at: http://www.google.com), the WHO Institutional Repository for Information Sharing (available at: http://www.who.int/iris), UNICEF (available at: http://www.unicef.int), and the WHO (available at: http://www.who.int).

Studies were included in the review if they focused on administration of >1 vaccine injection in a single visit to a child <1 year of age; only those studies that included IPV, PCV, or pentavalent vaccine were included for review. Four teams of 2 individuals reviewed unique abstracts, identified on the basis of the inclusion and exclusion criteria, for evidence on each of the following topics relevant to multiple-injection practices: (1) vaccine spacing, based on evidence for giving 2 injections 2.5 cm (ie, 1 inch) apart; (2) site of injection, based on evidence on the suitability of using the vastus lateralis (anterolateral thigh) versus other sites for intramuscular injections in infants; (3) route of injection, based on evidence favoring use of intramuscular delivery versus the subcutaneous route; and (4) procedural preparedness, based on guidelines for syringe- recapping procedures and preparation of multiple vaccines.

Information on the route of injection summarizes the actions taken by the GPEI to develop a communications strategy when initial concerns were raised from immunization program managers about increasing the number of recommended vaccine injections per visit during global IPV introduction in 2013–2015. This information is derived from the multiple authors who were involved in development of this communications strategy. For procedural preparedness, data on national immunization schedules from the WHO/UNICEF Joint Reporting Form [12] and GPEI IPV introduction [13] reporting mechanisms were analyzed for 2014 and 2015 to evaluate changes to the prevalence of recommended vaccine injections per visit across all countries. Based on each country’s national vaccination schedule submitted to the WHO and UNICEF, we calculated the number of countries globally and per WHO region that recommended a visit with delivery of 2, 3, 4, or 5 injectable vaccines in 2014 and 2015.

## RESULTS

### Provider and Caregiver Acceptance of Multiple Injections

With regard to acceptance of administering multiple injections at a single visit, a systematic review by Wallace et al found high caregiver acceptance of multiple injections in a single visit, even when the caregiver expressed concerns about the number of vaccine injections children received. Acceptance of all vaccine injections was markedly higher with a positive provider recommendation to the caregiver and high level of concern for the severity of the disease without vaccination [14]. These studies, almost all from higher-income countries, consistently found that providers significantly overestimated caregivers’ unwillingness to allow children to receive multiple injections at a single visit, indicating a need for evidence-based communications targeted toward providers. In addition, a report of a qualitative and quantitative assessment from South Africa demonstrated that, 5 years after adoption of an immunization schedule with 3 injectable vaccines at each of 2 infant immunization visits, 97% of caregivers were satisfied with the schedule and 97% of healthcare providers used the correct protocol [15]. Similar to the experience in high-income countries, recommendations from healthcare providers substantially impacted caregivers’ willingness to allow children to receive multiple injections. An unpublished report on a qualitative evaluation in Tanzania from 2015 documented that most service providers were comfortable with administering 3 injections at the same visit [16]. Caregivers and community leaders were accepting multiple injections for reasons such as the inability of infants to fear injections, reductions in cost and time owing to fewer visits, and protection of infants from vaccine-preventable diseases as early as possible. Pain caused by injections was expressed as a concern but was outweighed by the benefits of immunization.

### Available Evidence for Guidelines When Administering Multiple Injections

A preliminary systematic review of the safety and immunogenicity of administering IPV, PCV, and pentavalent vaccines during a single visit found that the vaccines were well tolerated and that there was a good immune response to each vaccine [10, 17]. Of the 1321 articles reviewed by title and abstract, 33 pertained to at least one multiple-injection practice of vaccine spacing, location of injection, site and route of injection, and or procedural preparedness.

#### Vaccine Spacing: Basis for the Recommendation of Giving 2 Injections 2.5 cm Apart

Official guidance in Australia, Canada, and the United States recommends a minimum distance of 2.5 cm between injections in the same limb; however, no published studies compared the spacing distances between injections (eg, 2.5 cm vs 3.5 cm) [18–20]. This recommendation is based on the practical necessity to differentiate local reactions from each vaccine, should they occur.

#### Site of Injection: Suitability of Vastus Lateralis Versus Other Sites for Intramuscular Injections in Infants

Five literature reviews and 11 clinical trials or observational studies addressed the suitability of administering intramuscular injections in the deltoid muscle versus the vastus lateralis muscle in infants. These papers concluded that accurate identification of the injection site and adequate muscle mass are crucial to avoid nerve and muscle injury [21–25]. Because of the small site and insufficient deltoid muscle mass in infants, there was agreement on limiting the potential number of injections administered by the deltoid route in a given healthcare encounter. Sources cited ages from 12 to 35 months as suitable ages to start deltoid intramuscular injections [20, 23–25].

The vastus lateralis muscle (located in the anterolateral region of the thigh) is the recommended intramuscular vaccination site for infants because its muscle mass is sufficient from birth, and study authors deemed it an appropriate site for children receiving multiple injections [21–26]. The risk for major injury was reported to be low because this area does not contain major nerves or blood vessels. Fibrosis and contracture have been the most commonly reported complications at this site [21, 22, 24–26]. The ventrogluteal (hip) site has been presented as a suitable alternative to the anterolateral thigh for infants and young children and has a low risk for injury [21, 22, 24, 25, 27]. Two recent randomized controlled trials in Brazil and Australia found little difference in immunogenicity, safety, or acceptability of vaccines administered in the anterolateral thigh versus hip sites in neonates, infants, and young children [28, 29].

It was noted that, at all injection sites, intramuscular injections can cause complications such as muscle contracture, nerve injury, and abscess formation; however, these are rare occurrences.

#### Route of Injection: Preference of the Intramuscular Versus the Subcutaneous Route of Vaccine Administration

Fourteen articles met our inclusion criteria for the review; of these, 5 were clinical trials or systematic reviews that presented relevant results. One review noted that the practice of intramuscular versus subcutaneous administration for vaccines has been based on tradition [30]. Two reviews identified that the majority of included studies found that injection site reactions were less likely and that immunogenicity was greater with intramuscular as compared to subcutaneous administration [30, 31]. One study noted that IPV administered subcutaneously caused local reactions in very few cases (ie, ≤3%) and that there was no difference in the frequency of local reactions between intramuscular and subcutaneous administration for IPV-containing vaccines [32]. A study on Hib combination vaccines administered simultaneously with diphtheria vaccines via the subcutaneous route did not identify any serious or persistent adverse reactions [33].

#### Procedural Preparedness: Syringe Recapping Procedures and Preparation of Multiple Vaccines

For a session with multiple injections, the Immunization Action Coalition (USA) recommends drawing up all of the vaccines indicated for 1 infant in a clean designated area, covering each clean needle with its cap, labeling each syringe, and then administering all the indicated vaccines to the infant in quick succession. While recapping used needles should be avoided to prevent needlestick injuries, unused needles have no risk of blood-borne pathogen exposure for the healthcare provider or the infant [34]. A 2015 document published by the WHO provides an alternative practice, stating, “Prepare each dose immediately before its administration—do not prepare several syringes in advance”; recapping needles should be avoided, but if absolutely necessary then a 1-hand scoop technique can be used to recap a clean needle [35]. Overall, many guidelines reviewed did not have clear instructions for a preferred vaccine preparation process, and we were unable to identify existing evidence to support one practice over another. An article by the Storage, Handling, Administration, and Preparation Experts’ Vaccine Delivery Working Group similarly indicated that vaccine preparation and administration is an area that has not been critically evaluated and that guidance varies among programs [36].

#### WHO SAGE Conclusions Regarding Visits Involving Multiple Injections

**Figure 2.**
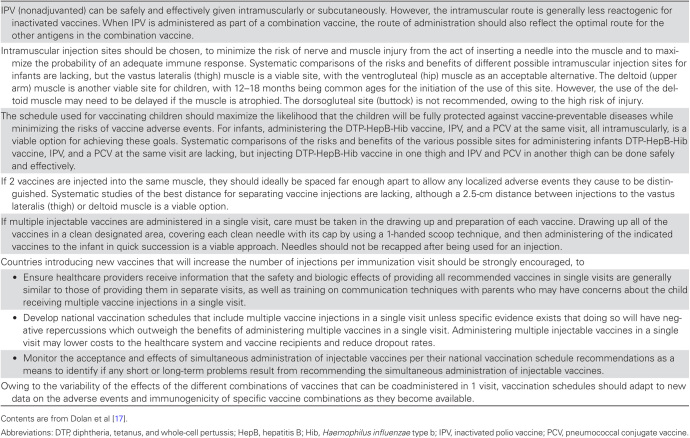
Key Conclusions and Recommendations on the Administration of Multiple Injectable Vaccines, Based on Findings of the Systematic Review Presented to the Strategic Advisory Group of Experts on Immunization, April 2015 [17]

The SAGE was presented with key conclusions and recommendations based on this review (Figure 2). SAGE members agreed that there was a lack of evidence on the topics concerning the distance between injection sites and the preparation procedure used for a multiple-injection visit and noted the need for additional research on topics surrounding the administration of multiple injections in a single visit [37]. They supported the coadministration of IPV, pentavalent vaccine, and PCV during a single visit, and they noted that evidence on immunogenicity and safety should be reviewed at the country level for other vaccine combinations administered simultaneously but specified that countries should not make modifications to their immunization schedules to prevent creation of multiple-injection visits. The SAGE also recommended that countries provide training to healthcare providers on both administration practices and communication strategies for vaccination sessions with multiple injections.

### Communication Messages on Vaccine Safety and Acceptability

The SAGE’s recommendations reinforced the messages of the communication materials developed by the GPEI for health sector staff on the benefits of multiple injections, the safety of IPV when coadministered with other vaccines, and the ability to respond to parental concerns about IPV and multiple injections. Several key messages were communicated by the GPEI. First, multiple injections are safe when given at 1 visit, based on experience from many countries in which ≥3 injections are administered during a single visit. Second, delaying vaccination for concerns of multiple injections delays the infant from being protected against potentially life-threatening diseases, leads to more vaccination visits for caregivers, and reduces the efficiency of immunization programs. Third, with 3 injectable vaccines (IPV, PCV, and pentavalent vaccine), IPV and PCV injections should be administered in one thigh, with the injection sites separated by at least 2.5 cm, and the pentavalent vaccine injection should be given in the opposite thigh because pentavalent vaccine is potentially the most reactogenic of the 3 vaccines [38]. These recommendations were also accompanied by evidence-based measures to reduce pain during vaccination, statements on the importance of good communication, and suggestions for responding to parental concerns and frequently asked questions. Brazil’s experience since introducing IPV nationwide in August 2012 as a third injection with pentavalent vaccine and PCV for children 2 and 4 months of age was informative for developing communications materials, particularly since first-dose IPV administrative coverage reached 80% in the fourth quarter among children aged <1 year [39].

Information was communicated through several strategies, including development of guidance and toolkits geared toward various audiences, such as decision-makers, technical staff, and vaccinators. Webinars and trainings for consultants and partners were held using evidence-based tool kits to address issues, concerns, and solutions related to multiple injections. All available information was broadly disseminated to partners, stakeholders, and countries. This strategy reinforced the evidence-based message on the safety and acceptability of multiple injections and emphasized the need for a strong healthcare provider endorsement of multiple injections, an essential means to increase caregiver acceptance of vaccines.

### Prevalence of Recommended Visits Involving >1 Vaccine Injection During the 2013–2015 Global IPV Introduction

Before 2013, 68 OPV-using countries had introduced IPV and 60 had done so using a combination vaccine that incorporated IPV alongside other antigens. Based on available data, of the 85 countries that introduced IPV during 2013–2015, 46 (55%) used standalone IPV and adopted a schedule with 3 injectable vaccines in a single visit, 26 (31%) used standalone IPV and adopted a schedule with 2 injectable vaccines in a single visit, 4 (4%) used standalone IPV and modified their pentavalent and/or PCV schedule to provide either IPV and 1 other injectable vaccine (PCV or pentavalent) in a single visit or only IPV in a single visit, and 9 (11%) used a combination vaccine and therefore avoided increasing the number of injections in a single visit [40]. In the WHO African Region and Region of the Americas, where 25 and 21 countries, respectively, introduced IPV during 2013–2015, approximately 70% of countries adopted a schedule with 3 injectable vaccines in a single visit (Table 1). Of the 43 remaining countries with planned IPV introductions in 2016 and 2017, 24 plan to adopt schedules with 3 injectable vaccines in a single visit. Over the course of the 2013–2017 IPV introduction into 126 countries, at least 70 countries (55%) either have adopted or plan to adopt schedules with at least 3 injectable vaccines recommended during the visit at which IPV is administered.

## DISCUSSION

Access to IPV for all countries is a key component of the GPEI endgame strategy for phasing out use of OPV, starting with the globally synchronized switch from tOPV to bOPV in April 2016. Increasing concerns regarding the administration of multiple injections at a single visit emerged as a potential cause of delays in IPV introduction and the polio endgame strategy. The logistics and communications issues associated with the addition of another injection to the primary immunization schedule were complex for countries and national technical advisory groups, particularly given the short timelines and the sometimes concurrent introduction of PCV.

Despite anticipated concerns over the acceptance of multiple injections, all 126 countries committed to introducing IPV as part of the endgame. By August 2016, 105 countries had introduced standalone IPV, and many had adopted schedules that included the administration of 3 injectable vaccines at a single visit [41].

Regional factors, potentially including differences in sensitivity to the costs of combination vaccines or additional immunization visits, cultural preferences regarding medical injections, or proclivities to follow WHO recommendations, may have influenced countries’ decisions to create visits with ≥3 injectable vaccines.

To date, assessments of acceptance of the administration of 3 injectable vaccines in a single visit in countries that introduced IPV in 2014–2016 have indicated that the vast majority of healthcare providers and caregivers accept such schedules. In the Gambia, all children with an immunization visit at 4 months of age received the recommended doses of IPV, PCV, and pentavalent vaccine when all 3 vaccines were in stock [42]. In Albania, 85% of children with immunization visits at 2 or 4 months of age received all of the recommended doses of IPV, PCV, and pentavalent vaccine at a single visit [43]. In these 2 cases and as previously found in South Africa, concerns about the addition of a new injectable vaccine dose for infants did not prevent high uptake of IPV, PCV, and pentavalent vaccine [44]. In contrast, early evaluations of Bangladesh’s introduction of IPV and PCV in March 2015 suggested that the creation of a new immunization visit for children at 18 weeks of age for the third dose of PCV, a change made to avoid administering >2 injectable vaccines to children at 14 weeks of age, may have led to lower uptake of the third dose of PCV than for IPV and the third dose of pentavalent vaccine given at 14 weeks of age [45].

Our systematic review of administration practices identified a need for comprehensive guidance for healthcare providers on preparing and administering multiple injections, as well as a need for research to support such guidance, as also documented by other studies [46]. Administration practices have been influenced by healthcare provider experience, tradition, and personal judgment, as well as by guidance that continues to shape practice despite a lack of evidence and specificity, such as the appropriate route of injection and the most-suitable injection sites [36, 47]. Systematic comparisons of the risks and benefits of various possible sites for administering pentavalent vaccine, IPV, and PCV to infants during the same visit are lacking and would be difficult to conduct, as would be studies of the most appropriate distance between vaccine injections administered in the same limb during the same visit.

Although several recommendations state that vaccines should be spaced at least 2.5 cm apart when administered in the same limb, no evidence was found to support this or any other distance as optimal for vaccine spacing in terms of reactogenicity or immunogenicity; however, this spacing has become standard practice among healthcare providers. This recommendation is based on the practical consideration that it allows for local reactions to each vaccine to be distinguished by providers. Additional reactogenicity information on other vaccine combinations is needed to provide guidance on whether some vaccines may need spacing of >2.5 cm.

Avoidance of the deltoid muscle for intramuscular injections in infants is based on the risk for nerve injury when infants have atrophied muscles [24]. Guidance documents recommend that use of this injection site can begin when children reach 12–35 months of age. Although administering vaccines intramuscularly versus subcutaneously is vaccine specific, based on our findings the intramuscular route less frequently causes adverse reactions for aluminum-adjuvanted, live attenuated, and nonadjuvanted/whole-cell vaccines. [30, 31] For IPV, administration via the subcutaneous and intramuscular routes provides comparable reactogenicity and immunogenicity.

Guidance on the preparation procedures for multiple-injection visits is inconsistent across sources. When guidance was provided, it was vague and based on the best practices for prevention of used-needle recapping and needlestick injuries. National guidelines on provider preparation for a multiple-injection visit are needed to ensure that procedures adequately protect the patient and provider, as well as ensure that the correct vaccines are administered and recorded.

There were several limitations with our analyses. Because of the variety and number of combinations of vaccines administered to an infant during a single visit, topics could not always be separated by type of vaccine. Very few studies were conducted in developing countries. Additionally, the findings of our literature review were focused on the effects of administering IPV, PCV, and pentavalent vaccines at the same visit. While we expect that administering other vaccine combinations would result in similar frequencies of adverse events, it is possible that administering other vaccine combinations at a single visit instead of at separate visits could increase the risk of adverse events following immunization. For example, administering inactivated influenza vaccine with either PCV or DTaP-containing vaccine to children at the same visit instead of separately has been found to be associated with a small increased risk for febrile seizures [48].

As additional new injectable vaccines are introduced, the number of vaccine injections given at a single visit may continue to rise. Health care providers should be given information on the safety and value of providing all recommended vaccines in a single visit to infants, and national vaccination schedules should maximize the likelihood that children will be fully protected against vaccine preventable diseases by including multiple injections in a single visit while minimizing the risks of vaccine adverse events.

**Figure 3.**
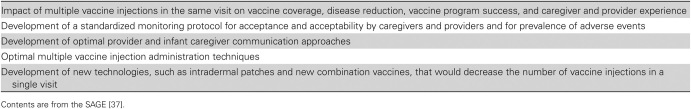
Key Research Topics and Activities on Multiple Vaccine Injections, According to the Advisory Group of Experts on Immunization (SAGE), April 2015

In summary, existing evidence supports the safety of administering IPV, pentavalent vaccine, and PCV at the same visit, as well as the acceptability of including multiple-injection visits in routine immunization schedules. A review of administration practices and techniques offers guidance to countries based on the little evidence available or long-standing experience and expert opinion. On the basis of this evidence, the SAGE recommended that countries provide training to healthcare providers on vaccine coadministration practices, including techniques to mitigate pain at the time of vaccination, information about safety and effectiveness of vaccines when coadministered, information about the likely overestimation of parental concerns, and training on improved communication strategies with caregivers. Additionally, the SAGE concluded that unless there is contrary evidence, countries should not prevent multiple vaccine injection visits from being included in recommended immunization schedules. Most countries that have introduced IPV since 2013 have included multiple-injection visits in their immunization schedules. With the inevitable introduction of more new vaccines into the EPI schedule, research and operational evaluations in low-income countries are critical to address issues related to pain management and improvement of efficiency during vaccination and to interventions to overcome provider and parental concerns about administering multiple injections in a single visit. The SAGE developed a recommended list of key research topics and activities (Figure 3). The multiple-injections experience in the context of the polio endgame emphasizes that anticipating the operational challenges of new policy statements can help achieve desired targets for new global health initiatives.

## Notes

***Acknowledgment.*** We thank Andrew Kroger for reviewing the manuscript.

***Disclaimer.*** The findings, interpretations, and conclusions expressed in this article are those of the authors and do not necessarily reflect the policies or views of the Centers for Disease Control and Prevention, the World Health Organization, or the Bill and Melinda Gates Foundation.

***Financial support.*** This work was supported by the Bill and Melinda Gates Foundation (grant OPP1095024 to M. P. and J. G.).

***Supplement sponsorship.*** This work is part of a supplement coordinated by the Task Force for Global Health with funding provided by The Bill and Melinda Gates Foundation and the Centers for Disease Control and Prevention.

***Potential conflicts of interest.*** All authors: No reported conflicts. All authors have submitted the ICMJE Form for Disclosure of Potential Conflicts of Interest. Conflicts that the editors consider relevant to the content of the manuscript have been disclosed.
